# Genetic and Molecular Regulation of Seed Storage Proteins (SSPs) to Improve Protein Nutritional Value of Oilseed Rape (*Brassica napus L.*) Seeds

**DOI:** 10.3389/fpls.2018.00890

**Published:** 2018-07-02

**Authors:** Katarzyna Gacek, Iwona Bartkowiak-Broda, Jacqueline Batley

**Affiliations:** ^1^Oilseed Crops Research Centre, Plant Breeding and Acclimatization Institute-National Research Institute, Poznań, Poland; ^2^School of Biological Sciences, The University of Western Australia, Crawley, WA, Australia

**Keywords:** *Brassica napus*, oilseed rape, seed protein, seed storage protein, genetic regulation

## Abstract

The world-wide demand for additional protein sources for human nutrition and animal feed keeps rising due to rapidly growing world population. Oilseed rape is a second important oil producing crop and the by-product of the oil production is a protein rich meal. The protein in rapeseed meal finds its application in animal feed and various industrial purposes, but its improvement is of great interest, especially for non-ruminants and poultry feed. To be able to manipulate the quality and quantity of seed protein in oilseed rape, understanding genetic architecture of seed storage protein (SSPs) synthesis and accumulation in this crop species is of great interest. For this, application of modern molecular breeding tools such as whole genome sequencing, genotyping, association mapping, and genome editing methods implemented in oilseed rape seed protein improvement would be of great interest. This review examines current knowledge and opportunities to manipulate of SSPs in oilseed rape to improve its quality, quantity and digestibility.

## Introduction

It has been estimated that the world global food demand will more than double by 2050, mainly due to the rapidly growing world population, increasing urbanization and changes in food preferences. Protein has been identified as the most limiting macronutrient, and sufficient protein quantity and quality will be required as the global food demand increases (FAO, 2013 ^1^). Oilseed rape (*Brassica napus*, canola) is the second largest oil producing crop in the world after soybean, and the by-product of the oil production is a protein rich meal or cake (OECD-FAO, 2015 ^2^; Oil World, 2017 ^3^). At present canola meal is primarily used in animal feed, but it can have application as a high quality food protein source, as well as in bio products such as formation of films, gels, foams, emulsions (Wanasundara et al., 2016).

The seeds of *B. napus* contain around 45% oil and 25% seed storage proteins (SSPs), which are predominantly composed of cruciferin (60%), napin (20%) and other minor proteins such as oleosins and lipid transfer proteins (Gehrig et al., 1996). When compared to soybean and legumes, the protein of rapeseed contains an excellent balance of essential amino acids, including high levels of sulfur containing amino acids (cysteine, methionine in napin), which are very desirable for human consumption, and a slightly limited amount of lysine. However, the introduction of modern varieties of oilseed rape with low glucosinolate (GLS) content lowered the amount of the desired napin in seeds. The pathways of amino acid and glucosinolate biosynthesis share common enzymes, therefore perturbation of glucosinolate in double low varieties could affect the level of napin (Field et al., 2004; Nesi et al., 2008). The level of napin and cruciferin in seeds also affects the functionality of canola protein products, including solubility, emulsifying ability and heat induced gel formation. Both cruciferin and napin are soluble above pH 5.5, whereas napin is also soluble at a lower pH 3-4. Napin exhibits poorer emulsifying abilities, therefore cruciferin rich oilseed rape proteins show higher emulsion capacities. Cruciferin has also stronger tendency to form heat-induced gels when compared to napin (Wanasundara et al., 2016). Various industrial purposes require different level of cruciferin and napin in the seed proteins of *B. napus*.

Current goals in oilseed rape meal improvement is to make it a useful protein source for non-ruminants, especially for poultry nutrition (Wanasundara et al., 2016). For this it is necessary to reduce the most limiting anti nutritional factors, such as glucosinolates, phytic acid, fiber, sinapates and non-starch polysaccharides (NSP). Sinapates have a negative effect on the smell of the eggs therefore their use is limited for laying hens. Phenolic compounds, such as sinapates, flavonoids and lignins are present in the maternally derived seed coat tissue and play an important role in pigmentation and protection against various environmental injuries (Qu et al., 2016). In Arabidopsis several genes regulating phenolic compounds named *TRANSPARENT TESTA* (*TT*) were identified, and the seeds of the mutants defective in flavonoids accumulation are pale yellow to pale brown (Johnson et al., 2002). *TT8* was also found to regulate activity of the four master regulator genes and inhibits seed fatty acid accumulation (Chen et al., 2014). In oilseed rape yellow seeded genotypes derived through breeding are deprived of tannins, contain lower level of fiber, higher level of protein content but their digestibility is not improved (Swiech et al., 2016). The yellow seeded genotypes are advantageous to incorporate into genomic studies in order to identify genetic variation underlying the important trait of interest. Current challenges would also include providing consistent quality of protein meal on the market. There is also a great interest to improve oilseed rape meal for human consumption. It would mean elevating the content of proteins in the seeds and manipulating the ratio of napins/cruciferins in the composition of the proteins within *B. napus* seeds. As oilseed rape is mainly used for animal feed, the knowledge of its nutritional value for humans is quite limited. The digestibility of oilseed rape protein was found to be similar to that of soy protein (Bos et al., 2007). The analysis of oilseed rape protein products (Isolexx) showed a low level of anti nutritional factors which make them good candidates for a novel food ingredient (EFSA NDA Panel [EFSA Panel on Dietetic Products and Nutrition and Allergies], 2013). The studies of oilseed rape peptide mixtures have proved their role in lowering the blood pressure of hypertensive mice and showed their antioxidant, antidiabetic, anorexigenic, anticancer, antiviral, hypercholesterolemic and bile acid binding activities (Wanasundara, 2011; Aachary and Thiyam, 2012; Alashi et al., 2013).

In this review we will look into current knowledge in genetic regulation of biosynthesis and accumulation of SSPs in the model species Arabidopsis, Brassicas and other crop species including maize, beans, pea, broad beans and *Vicia narbonensis*. We will also summarize recent advances in genomic studies related to seed protein content in *B. napus* and show the prospects to improve the quality and quantity of protein in this crop species for animal feed, human consumption and industrial purposes.

## Seed Storage Proteins of *B. napus*

The SSPs, together with oil and starch, form seed storage reserves which accumulate in the matured embryo of *B. napus* seed and supply energy for seed germination, growth, as well as provide energy for humans and animals. The analysis of spatial and temporal napin, cruciferin and oleosin accumulation revealed that their storage during early and middle stages of seed development was mostly restricted to the radicle of the embryo. By the late stage of seed growth the accumulation of SSPs ceased. The pattern of SSPs accumulation corresponded with that of lipids which indicates that the two compete for substrate, energy and space (Borisjuk et al., 2013).

The identified in Arabidopsis cruciferin encoding genes were named *CRUCIFERINA1, CRUCIFERINA2, CRU3/CRUCIFERIN C*, and *CRUCIFERIN B* and *2S* precursors (*2S1–2S5*) (Gruis et al., 2002, 2004). Genes encoding cruciferin precursors have been also identified in *B. napus* and comprise of a five-member gene family (Breen and Crouch, 1992) (**Table 1**). Napin in *B. napus* is also encoded by multi-gene families (Josefsson et al., 1987; Scofield and Crouch, 1987; Baszczynski and Fallis, 1990; Sjodahl et al., 1991) and in order to manipulate the composition of SSPs in oilseed rape to a more desirable level of napin, genetic engineering studies have been performed in this crop species (Altenbach et al., 1992; Hannoufa et al., 2014). Introduction of the antisense gene for cruciferin resulted in increased levels of cysteine, lysine and methionine essential amino acids in the SSPs of the *B. napus* seeds. Downregulation of napins in oilseed rape resulted in increased level of cruciferins and lower amounts of cysteine and lysine (Guerche et al., 1990; Kohnomurase et al., 1994, 1995). Despite the genetic engineering studies, genomic studies are desired to identify key genes regulating napin content in the SSPs of oilseed rape in order to introduce that genetic variation into the modern, double low varieties of oilseed rape.

**Table 1:** List of genes identified in previous studies, known to be involved in accumulation, synthesis and transcriptional regulation of SSPs.

*Arabidopsis thaliana* gene	Gene name	Gene function	Reference
**SSPs**			
AT5G44120	*CRUCIFERIN 1*	Response to abscisic acid, seed maturation	Gruis et al., 2002, 2004
AT1G03880	*CRUCIFERIN2*	Response to abscisic acid, seed maturation	Gruis et al., 2002, 2004
AT4G28520	*CRUCIFERIN 3*	Response to abscisic acid, seed maturation	Gruis et al., 2002, 2004
**Accumulation of SSPs**			
AT1G58360	*AMINO ACID PERMEASE 1*	Amino acid transport	Rolletschek et al., 2005; Gotz et al., 2007; Weigelt et al., 2008; Sanders et al., 2009
AT5G09220	*AMINO ACID PERMEASE 2*	Amino acid transport	Zhang et al., 2010
AT5G04770	*cationic amino acid transporter*	Amino acid transport	Hammes et al., 2006
AT1G10010	*AMINO ACID PERMEASE 8*	Amino acid transport	Schmidt et al., 2007
AT2G02040	*PEPTIDE TRANSPORTER 2*	Peptide transport	Song et al., 1997
**Synthesis of SSPs**			
AT3G52850	*VACUOLAR SORTING RECEPTOR 1*	Protein targeting to vacuole, protein transport	Zouhar et al., 2010
AT2G30290	*VACUOLAR SORTING RECEPTOR 2*	Protein targeting to vacuole, protein transport	Zouhar et al., 2010
AT2G14740	*VACUOLAR SORTING RECEPTOR 3*	Protein targeting to vacuole, protein transport	Zouhar et al., 2010
AT2G14720	*VACUOLAR SORTING RECEPTOR 4*	Protein targeting to vacuole, protein transport	Zouhar et al., 2010
AT5G66160	*RECEPTOR HOMOLOGY REGION TRANSMEMBRANE DOMAIN RING H2 MOTIF PROTEIN 1 (RMR1)*	Intracellular protein transport	Park et al., 2005
AT3G54300	*VESICLE-ASSOCIATED MEMBRANE PROTEIN 727*	Protein targeting to vacuole	Ebine et al., 2008
AT5G46860	*SYNTAXIN OF PLANTS 22*	Intracellular protein transport	Ebine et al., 2008
AT5G39510	*VESICLE TRANSPORT V-SNARE 11*	Protein targeting to vacuole	Ebine et al., 2008
AT1G16240	*SYNTAXIN OF PLANTS 51*	Intracellular protein transport	Ebine et al., 2008
AT1G54370	*NA + /H + ANTIPORTER 5*	Lithium ion transport, potassium ion transmembrane transport, regulation of intracellular pH	Wu et al., 2016
AT1G79610	*NA + /H + ANTIPORTER 6*	Lithium ion transport, potassium ion transmembrane transport, regulation of intracellular pH	Wu et al., 2016
***Transcriptional and hormonal regulation of SSPs accumulation***			
AT1G21970	*LEAFY COTYLEDON 1*	Transcription factor	Kroj et al., 2003; Kagaya et al., 2005
AT1G28300	*LEAFY COTYLEDON 2*	Transcription factor	Kroj et al., 2003; Kagaya et al., 2005
AT3G26790	*FUSCA3*	Transcription factor	Wang et al., 2007
AT3G24650	*ABSCISIC ACID INSENSITIVE 3*	Transcription factor	Kroj et al., 2003; Kagaya et al., 2005
AT4G00480	*MYC1*	Transcription factor basic helix-loop-helix (bHLH)	Fernandez-Calvo et al., 2011; Gao et al., 2016
AT1G32640	*MYC2, JASMONATE INSENSITIVE 1*	Transcription factor	Fernandez-Calvo et al., 2011; Gao et al., 2016
AT5G46760	*MYC3*	Transcription factor	Fernandez-Calvo et al., 2011; Gao et al., 2016
AT4G17880	*MYC4*	Transcription factor	Fernandez-Calvo et al., 2011; Gao et al., 2016
AT3G17860	*JASMONATE-ZIM-DOMAIN PROTEIN*	Transcription factor	Fernandez-Calvo et al., 2011; Gao et al., 2016

## Genetic Regulation of Synthesis and Accumulation of SSPs During Seed Growth and Development

**FIGURE 1:**
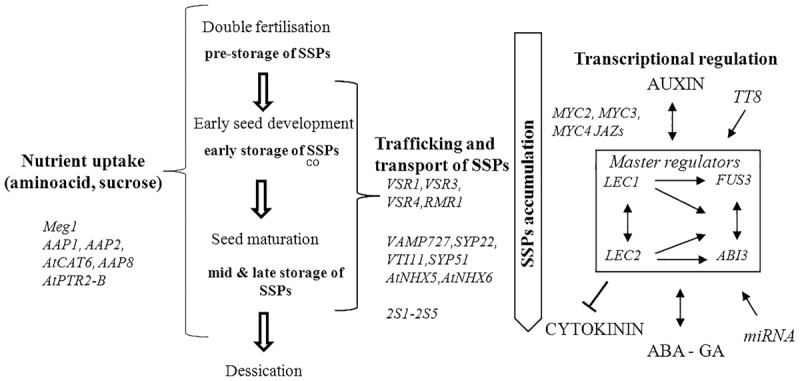
Summary of genetic and transcriptional regulation of seed storage proteins (SSPs). Accumulation of SSPs occurs from early to late storage stage of seed development; en, endosperm. The genes regulating SSPs in seed consist of genes identified during nutrient uptake, trafficking and transport of SSPs. SSPs accumulation is also orchestrated by the regulatory network of transcription factors (master regulators), hormones, miRNA and other genes during seed development. The interactions of genes are depicted with arrows and T-bar.

The majority of genes responsible for regulation of SSPs synthesis and accumulation in seeds have been identified in genetic studies using model species *Arabidopsis thaliana*. High collinearity between *A. thaliana* and *B. napus* genomes is a great advantage therefore the identified genes are great candidates to study in *B. napus* (**Figure 1** and **Table 1**).

### Synthesis of SSPs

Seed storage proteins are synthesized in the endoplasmic reticulum (ER) and transported to protein storage vacuoles (PSV) where they are primarily accumulated. The trafficking of the mature SSPs are transported in the Golgi dependent and independent manner (Hohl et al., 1996; Hara-Nishimura et al., 1998). Several vacuolar sorting receptors (VSR) genes including VSR1, VSR3, VSR4 and RMR1 have been identified in this process in Arabidopsis (Park et al., 2005; Zouhar et al., 2010). VAMP727, SYP22, VTI11, and SYP51 compose the SNARE complex which mediates fusion between PVC and vacuole (Ebine et al., 2008). AtNHX5 and AtNHX6 regulate localization of the SNARE complex and thus its function in protein transport (Wu et al., 2016). The genes are listed in **Table 1** and **Figure 1**.

### Accumulation of SSPs

Accumulation of seed storage reserves is highly coupled with seed growth and development (**Figure 1**). Seed growth is initiated with a process of double fertilization, when one sperm cell fertilizes an egg cell to generate a diploid embryo and a second sperm cell fuses with the central cell giving rise to the triploid endosperm. Embryo and endosperm are surrounded by maternal integuments, future seed coat. In the early stages of development, organic nutrients in the form of sucrose, amino acids and potassium are imported from the maternal tissue to the seeds via vascular tissue within the funiculus which terminates in the chalazal part of the seed coat. Many genes involved in the transport of sugar, amino acids, lipids and hormonal regulation were found to be specifically expressed in this part of the seed, which indicates its importance in seed filling (Millar et al., 2015). Released nutrients are mainly accumulated in the endosperm, which acts as a nourishment to the developing embryo. In maize, genes specifically expressed in the region of endosperm which surrounds the embryo (*ESR* genes) were found to regulate nutrient transfer to the embryo (Bonello et al., 2000, 2002). *Meg1* genes identified in the endosperm of maize seeds ensure maternal nutrient uptake, sucrose partitioning and seed biomass yield in maize (Costa et al., 2012). In the endospermic seeds, such as Arabidopsis and Brassica, the nutrients are also thought to be released from the seed coat by as yet unknown transport proteins. In *Vicia faba*, *Pisum sativum*, *Vicia narbonensis*, and *A. thaliana* Amino acid permase *AAP1* mediates uptake of amino acids by the embryos and thus regulates SSP accumulation and seed yield (Rolletschek et al., 2005; Gotz et al., 2007; Weigelt et al., 2008; Sanders et al., 2009).

In Arabidopsis another amino acid permase *AAP2* and cationic amino acid transporter *AtCAT6* were also shown to play a role in supplying amino acid and higher SSP accumulation in seeds (Hammes et al., 2006; Zhang et al., 2010). Amino acid permases *AAP8* was identified with a role in the uptake of amino acids into the endosperm during early embryogenesis (Schmidt et al., 2007). Peptide transport gene, *AtPTR2-B* was shown to play a role in general plant nutrition and seed development (Song et al., 1997). The translocation of amino-N and loading process of amino acids in the phloem sap of leaves affect protein content in the seeds of the Brassica species (Lohaus and Moellers, 2000; Tilsner et al., 2005). At maturity the endosperm of brassicas remains as a one cell layer and together with the cells of embryo are packed full of PSV and oil bodies (Herman and Larkins, 1999). The orthologous genes identified to play a role in SSP accumulation in here mentioned crop species and model Arabidopsis have not been investigated in oilseed rape. These genes are excellent candidate genes to investigate in this crop in seed protein improvement programs. The genes are listed in **Table 1**.

### Transcriptional and Hormonal Regulation of SSPs Accumulation

Transcriptional regulation of the accumulation of SSPs in seeds occurs during the maturation phase of the seed. In Arabidopsis four ‘master regulator’ transcription factors (TFs) *LEC1, LEC2, FUS3*, and *ABI3* specifically expressed in seeds are known to play a role in seed maturation, accumulation of SSPs and controlling expression of many other regulators of metabolic pathways (Parcy et al., 1994; Verdier and Thompson, 2008). *LEC1* controls the expression of the SSP genes in a hierarchical manner, which involves *ABI3* and *FUS3* (Kroj et al., 2003; Kagaya et al., 2005). In Arabidopsis, *FUS3* was found to be associated with the induction of genes encoding SSPs: napin and cruciferin as well as enzymes involved in FA biosynthesis (Wang et al., 2007). The seeds of the loss of function mutant of *B. napus*, *BnFus3* showed decreased level of total seed oil content while seed protein content was increased. *BnFus3* mutant seeds showed lower expression level of genes involved in sucrose photo-assimilation and glycolysis, which might have affected the biosynthesis of both protein and oil and can explain their altered level in the seeds (Elahi et al., 2015). Overexpression of *BnLEC1* in oilseed rape enhanced seed oil production but did not affect the level of seed protein content (Tan et al., 2011). Other identified TFs which act in the accumulation of SSPs include basic helix-loop-helix (bHLH) MYC2, MYC3, and MYC4 which interact with JASMONATE ZIM-DOMAIN (JAZs) (Fernandez-Calvo et al., 2011; Gao et al., 2016). Recent studies allowed identification of miRNAs which regulate expression of TFs during seed growth and maturation (Gupta et al., 2017). In beans, a TF *ROM1* was found to modulate transcription from lectin and storage protein genes (Chern et al., 1996).

Regulation of seed storage accumulation is coupled with complex interplay of hormones with TFs (Verdier and Thompson, 2008). Abscisic acid (ABA) is key regulator of gene expression in the maturing seeds (Delisle and Crouch, 1989) and interacts with the four master regulator genes (Kagaya et al., 2005). In *B. napus*, the level of SSPs (cruciferin and napin) is higher when induced by abscisic acid (Delisle and Crouch, 1989). Protein phosphatase *ABI1* which regulates abscisic acid responses in plants was found to regulate the expression of napin genes in *B. napus* (Rask et al., 1998; Ezcurra et al., 2000). *LEC2* interplays with auxin and induces auxin responsive genes and downregulates cytokinin (Stone et al., 2008). *LEC1* also interacts with auxin (Casson and Lindsey, 2006) whereas *FUS3* acts as a negative regulator of gibberellic acid biosynthesis. The genes known to be involved in transcriptional regulation of SSPs are listed in **Table 1**.

## Genomic Studies in Seed Storage Protein in *B. napus*

Genetic mapping is an effective way to dissect the genetic mechanisms regulating important agronomic traits at the whole genome level. *B. napus* L. has a complex tetraploid genome AACC, 2n = 38 as it originated from spontaneous hybridization between two diploid species: *B. rapa* (AA, 2n = 20) and *B. oleracea* (CC, 2n = 18). Since oil is of a major economic interest in this crop species, the majority of QTL genetic mapping studies in *B. napus* so far have been performed to identify markers for seed oil content and only a few of such studies were conducted to unravel the genetic regulation of seed protein content in this crop. The results of those studies have shown that QTL for seed oil and protein content are closely linked and that there is a negative correlation between protein and oil content (Grami et al., 1977; Gül et al., 2003). These findings are not surprising as both protein and oil compete for the same basic substrates in the biochemical pathway and therefore must be partly controlled by the same genes. To unravel this problem, Zhao et al. (2006) applied a conditional mapping approach which allowed them to analyze the influence of genetic variation on protein content in oilseed rape seeds independently from oil content. The conditional QTL mapping allowed detection of five QTL for protein synthesis on separate linkage groups from oil content localized on chromosome A07, A09, C01, C08, C09 (**Table 2**) (Zhao et al., 2006). Another QTL genetic mapping study allowed identification of major QTL for protein content in oilseed rape seeds on chromosome A07 (Wurschum et al., 2012). Three QTL for napin content was detected on linkage groups A2, C6, C9 and two QTL for cruciferin were detected in linkage group A2 and C19 (Schatzki et al., 2014). Due to low mapping resolution and lack of marker sequence information, integration of these QTL onto the physical map and identification of underlying candidate genes was impossible.

**Table 2:** Summary of genetic mapping studies and list of the identified QTL for protein content in *B. napus* seeds.

Population	Number of markers	QTL number	QTL location	Candidate genes	Reference
Sollux x Gaoyou (284 DH lines)	125 SSR	5	A07, A09, C01, C08, C09	No	Zhao et al., 2006
391 DH lines from nine crosses among 10 parental lines	253 SNP	1	A07	No	Wurschum et al., 2012
Express 617 x R 53 (229 DH lines)	229 markers: 80 SSR and 149 AFLP	5	A2, C6, C9; A2, C9	No	Schatzki et al., 2014
Sansibar x Oase (226 DH lines)	1686 SSR and AFLP	4	A01, A07, C03	No	Teh and Mollers, 2016
KenC-8 x N53-2 (300 DH lines)	3207: 3106 SNP and 101 SSR STS	38	A02, A03,	BnaA03g38500D	Chao et al., 2017
			A04, A07,	BnaA04g01950D	
			A09, C01,	BnaA04g19410D	
			C03, C05,	BnaA09g02110D	
			C06, C07,	BnaA09g08190D	
			C08, C09	BnaA09g13220D	
				BnaA09g11520D	
				BnaC03g65080D	
				BnaC05g02160D	
				BnaC05g44560D	
				BnaC05g44510D	
				BnaC06g18820D	
				BnaC08g09910D	

The recent release of the oilseed rape reference genome (Darmour cultivar) (Chalhoub et al., 2014) and high resolution sequencing technologies (Edwards and Batley, 2010; Edwards et al., 2013) allow more accurate genetic mapping studies which lead to identification of potential candidate genes regulating the trait of interest. The high collinearity between *B. napus* and Arabidopsis is a great advantage as a lot of genes which affect biosynthesis and accumulation of SSPs in seeds have been already identified in this model plant and other crop species. Orthologous genes mapped in genetic mapping studies on protein content in *B. napus* seeds would be of interest. Teh and Mollers (2016) identified four minor QTL for protein content of defatted meal on chromosomes A01, A07 and C03 (**Table 2**). The identified QTL had the largest effect but no candidate genes were elucidated in this study. Interestingly, the most interesting hotspot of QTL were located on chromosome A01 which were linked to palmitic, oleic and linoleic fatty acids, oil content and protein content. The variation in this region led to decrease of palmitic, linoleic fatty acids and protein content in the defatted meal which led to increase of oil content in the seed (Teh and Mollers, 2016). Recently, the application of the 60K SNP Infinium Array allowed identification of 3700 single nucleotide polymorphisms (SNPs) in 38 QTL regions for seed protein content in *B. napus* (Chao et al., 2017). The potential candidate genes identified in this study encode seed storage 2S, caleosin, oleosin, and cruciferin. To date, this is the most accurate genetic mapping of protein content in *B. napus* which allowed identification of candidate genes involved in regulation of protein content in seeds of oilseed rape (**Table 2**).

## Summary and Future Prospects

As the world population increases, the demand of protein for animal feed and human consumption in the world will also be rising. Oilseed rape meal is a great source of plant protein but still requires improvement of its quality and quantity. As the quantity of seed protein is highly interconnected with oil content, therefore enhanced protein content should not affect the level of the primary desired seed oil. It is of great interest to introduce desirable traits present in the yellow seeded genotypes (less fiber, higher protein content), improve the level of lysine, reduce anti nutritional factors and engineer the oleosins to produce valuable proteins. Future prospects of improvement of oilseed rape as a protein crop would be integration of recent advances in genomic studies and bioinformatics methods to identify key genes regulating quantity of seed protein and its amino acids composition to implement them as molecular markers into marker assisted breeding. Recent advances in genome sequencing technology, knowledge of the oilseed rape reference genome together with the latest bioinformatics methods (Edwards et al., 2013; Glaubitz et al., 2014; Bayer et al., 2015) allow identification of high resolution sequence based markers which are very useful in genome wide association studies (GWAS). GWAS studies have already been proved to be a very useful tool in genetic dissection of agronomically important traits in *B. napus* (Pasam et al., 2012; Gacek et al., 2017). Implementation of the identified markers in GWAS or QTL studies into breeding practice includes validation of the genetic variant linked to a trait of interest with available molecular methods. One of such methods includes targeting of the identified genes with latest genome editing methods such as CRISPR/Cas9 technology (Bortesi and Fischer, 2015), which are great future prospects for breeding. The validated marker can only then become a functional genetic marker. Standard PCR based genotyping methods are then used to screen the pre-breeding material for identification of the desired genetic variant which allows selection of the most promising genotypes for further breeding programs. This can shorten the length of the breeding cycle of a new variety by a few years. For this reason integration of latest bioinformatics, sequencing technology methods and genetic mapping studies should be applied in breeding programs of seed protein quantity and quality in oilseed rape.

## Author Contributions

KG developed the idea and wrote the manuscript. IB-B and JB edited the manuscript and contributed ideas and suggestions.

## Conflict of Interest Statement

The authors declare that the research was conducted in the absence of any commercial or financial relationships that could be construed as a potential conflict of interest.

## Footnotes

1 http://www.fao.org/docrep/007/y5019e/y5019e03.htm

2 http://www.fao.org/fileadmin/templates/est/COMM_MARKETS_MONITORING/Oilcrops/Documents/Food_outlook_oilseeds/FO_May_2015.pdf

3 https://www.oilworld.biz/
